# Comparison of Bone Mineral Density in Lumbar Spine and Fracture Rate among Eight Drugs in Treatments of Osteoporosis in Men: A Network Meta-Analysis

**DOI:** 10.1371/journal.pone.0128032

**Published:** 2015-05-26

**Authors:** Ling-Xiao Chen, Zhi-Rui Zhou, Yu-Lin Li, Guang-Zhi Ning, Tian-Song Zhang, Di Zhang, Shi-Qing Feng

**Affiliations:** 1 Department of Orthopaedics, Tianjin Medical University General Hospital, 154 Anshan Road, Heping District, Tianjin, People’s Republic of China; 2 Department of Radiation Oncology, Fudan University Shanghai Cancer Center, Department of Oncology, Shanghai Medical College, Fudan University, Shanghai, 200032, China; 3 Internal medicine of traditional Chinese medicine department, Jing 'an district central hospital of Shanghai, NO. 259, Xikang road, 200040, Shanghai, P.R. China; Van Andel Institute, UNITED STATES

## Abstract

**Context:**

The preferred treatment for osteoporosis in men is debated, and pairwise meta-analysis cannot obtain hierarchies of these treatments.

**Objective:**

The objective of this study was to integrate the evidence and provide hierarchies of eight drugs based on their effect on the bone mineral density in the lumbar spine (BMD in LS) and the fracture rate.

**Data Sources:**

Eligible studies were identified by searching Amed, British Nursing Index, EMBASE, PubMed, the Cochrane Central Register of Controlled Trials (CENTRAL), Google Scholar, SIGLE, the National Technical Information Service, the National Research Register (UK), and the Current Controlled Trials databases.

**Study Selection:**

RCTs or quasi-RCTs reporting at least two drugs (two active drugs or one active drug and a placebo) used to treat osteoporosis in men were selected by two authors.

**Data Extraction:**

Two authors independently extracted the data.

**Data Synthesis:**

Thirteen studies involving 3647 patients were included. Compared with placebo therapy, zoledronate (SMDs 13.48, 95% credible intervals 11.88-15.08) yielded the most significant effect on increasing the BMD in LS, followed by alendronate (11.04, 9.68-12.41), teriparatide (20mcg) + risedronate (10.98, 8.55-13.48), risedronate (10.33, 8.68-12.01), teriparatide (20mcg) (9.33, 6.87-11.76), strontium ranelate (8.88, 7.51-10.24), ibandronate (5.49, 3.82-7.16), parathyroid hormone (1-84) (4.89, 3.12-6.62) and alfacalcidol (3.42, 1.7-5.2). Placebo therapy had a significantly higher fracture rate in contrast to risedronate (OR 2.51, 95% CrI 1.23-4.24) or zoledronate (2.92, 1.29-5.62) or teriparatide (20mcg) (4.04, 1.36-8.49) or teriparatide (40mcg) (3.5, 1.14-8.34). Zoledronate ranked first for increasing the BMD in LS, and teriparatide (20mg) was ranked first for decreasing the fracture rate.

**Conclusions:**

Zoledronate might be the best choice to increase the BMD in LS and teriparatide (20mg) might lead to the lowest fracture rate.

## Introduction

Osteoporosis is a common disease that impairs bone mass and bone microarchitecture and is a major cause of fragility fracture [1]. The fracture rate varies in different countries, and there has been a trend for the occurrence of fractures to decline in recent years [2]. Studies with a long follow-up duration, demonstrate that osteoporotic fractures lead to an increase in death, destitution and debility [3]. Several cohort studies indicate that improvement in the bone mineral density (BMD) reduces the osteoporotic fracture rate, although discrepancies in the literature also exist [4, 5].

Anti-resorptive drugs and bone-anabolic drugs are two main classes of the drugs to treat osteoporosis. Anti-resorptive drugs mainly include bisphosphonates, raloxifene and strontium ranelate, and the latter two are suggested for use in women with postmenopausal osteoporosis. Bisphosphonates (e.g., alendronate, risedronate, ibandronate and zoledronate) are widely used in postmenopausal women, men, and those with steroid-induced osteoporosis or with as Paget’s disease because of their high affinity for bone, low costs and safety [6, 7]. Parathyroid hormone (PTH 1–84) and teriparatide (PTH 1–34) belong to the bone-anabolic drug class as they can build up new bone, and the duration of their use is limited to 24 months due to the safety concerns [7–9]. Some randomized controlled trials (RCTs) have proved that alfacalcidol which is an active form of vitamin D is an effective and safe drug to treat osteoporosis [10, 11]. However, debates exist as to which therapy should be used first.

We aimed to compare the BMD in the lumbar spine (LS) and the fracture rate in osteoporotic men being treated with eight drugs (alfacalcidol, alendronate, ibandronate, risedronate, zoledronate, strontium ranelate, teriparatide and parathyroid hormone). Our intention was to provide hierarchies of the comparative BMD in LS and the fracture rate of the drugs. Therefore, a network meta-analysis was performed.

## Methods

### Criteria for considering studies

Studies were considered acceptable for inclusion in the meta-analysis if they met the following criteria: (1) Participants: Men with primary or idiopathic osteoporosis. For some studies including hypogonadal men, considering the number of these patients was small which had limited effect on the results, we also included these studies; (2) Interventions and comparisons: Therapy regimens that included two of the following drugs or one drug and a placebo: alfacalcidol, alendronate, ibandronate, risedronate, zoledronate, strontium ranelate, teriparatide and parathyroid hormone; (3) Outcomes: the BMD in LS (we chose the BMD in LS because the number of the studies reported LS was the largest. With the largest number of included patients, the results based on LS was more reliable than the results from the other sites) and the fracture rate; (4) Study design: randomized controlled trials (RCTs) or quasi-RCTs.

Trials were excluded if: (1) they were abstracts, letters, or meeting proceedings; (2) they contained repeated data or did not report the outcomes of interest; or (3) the duration of follow-up was < 12 months.

### Search methods and study selection

We searched Amed (from 1985 to May 2014), British Nursing Index (from 1985 to May 2014), EMBASE (from 1974 to May 2014), PubMed (from 1966 to May 2014), the Cochrane Central Register of Controlled Trials (CENTRAL) (The Cochrane Library, most recent issue), Google scholar, SIGLE (System for Information on Grey Literature in Europe) and clinicaltrials.gov. Keywords and MeSH terms including “alfacalcidol”, “alendronate”, “ibandronate”, “risedronate”, “zoledronate”, “strontium ranelate”, “teriparatide”, “parathyroid hormone”, “men or male” and “osteoporosis” were used in the search strategy. We performed a primary search pertaining to vitamin D and osteoporosis in PubMed and found no relevant RCT involving vitamin D and osteoporosis in men except for alfacalcidol. So we searched other databases for studies using alfacalcidol. We also viewed the reference list of the included studies for any additional papers. We included only articles written in English.

Two authors independently made the selection based on the title and abstract. Any disagreement between the two authors was resolved by a discussion. If there was no consensus, a third reviewer (Feng) was consulted.

### Data extraction and assessment for risk of bias

Information including trial name, sample size, comparators, country, clinical setting and maximum follow-up time were extracted by the two authors for each included study. Dichotomous data were used for reporting the fracture rate. The fracture rate was referred to as the incidence of new vertebral fractures, which was determined by radiograph of the vertebral column. The fracture rate was reported as odds ratios (ORs) with a 95% confidence interval (CI) for direct comparisons or 95% credible intervals (CrI) for indirect comparisons. For continuous data (e.g., BMD in LS), the standardized mean differences (SMDs) with a 95% CI for direct comparisons or CrI for indirect comparisons were used. We contacted the first or corresponding author of the included trials to obtain any missing information. We used the Cochrane risk of bias tool to assess the risk bias of the included studies [12]. The tool included seven domains that included random sequence generation, allocation concealment, blinding of participants and personnel, blinding of outcome assessment, incomplete outcome data, selective reporting and other bias (funding and baseline imbalance). The judgment for each domain was a low risk of bias (sufficient information to describe the right methods), a high risk of bias (sufficient information to describe the wrong methods), or an unclear risk of bias (insufficient information to describe the methods) and two authors independently evaluated the risk of the studies.

### Data synthesis and analysis

Two outcomes (BMD in LS and fracture rate) were analyzed. To evaluate whether there was inconsistency between direct and indirect evidence, we compared the pooled ORs or SMDs calculated from the network meta-analysis with the corresponding effect size from pair-wise meta-analysis. At first, we made pairwise meta-analyses for studies that directly compared different treatments using Stata software (version 12.0, StataCorp, College Station, TX). The DerSimonian and Laird random effects model was used. Chi-square tests and I-square tests were used for testing heterogeneity between studies. For publication bias, we would use the funnel plots if the number of included studies in one pair of comparison was larger than 10. Then, network meta-analysis was performed using WinBUGS (version 1.4.3, MRC Biostatistics Unit, Cambridge, UK) with random effects models developed by Chaimani (downloaded from www.mtm.uoi.gr). For the network meta-analysis, the posterior parameters were calculated by Markov chain Monte Carlo methods. Non-informative uniform and normal prior distributions were performed to fit the model [13]. An automatically generated starting value was used to fit the model [13]. For each analysis, we used 300,000 iterations after an initial burn-in of 50,000 [14]. To rank the treatments, we used the surface under the cumulative ranking probabilities (SUCRA) to indicate which treatment was the best one [15]. Finally, the robustness of the model was tested by calculating the posterior mean residual deviance [16] with R (version 3.1.1, R Foundation for Statistical Computing, Vienna, Austria). When the posterior mean residual deviance approximated the data points, the model fit the data well. Sensitivity analyses were performed by excluding studies with a high risk of bias.

There was no protocol.

## Results

### Study selection and characteristics of included studies

**Fig 1:**
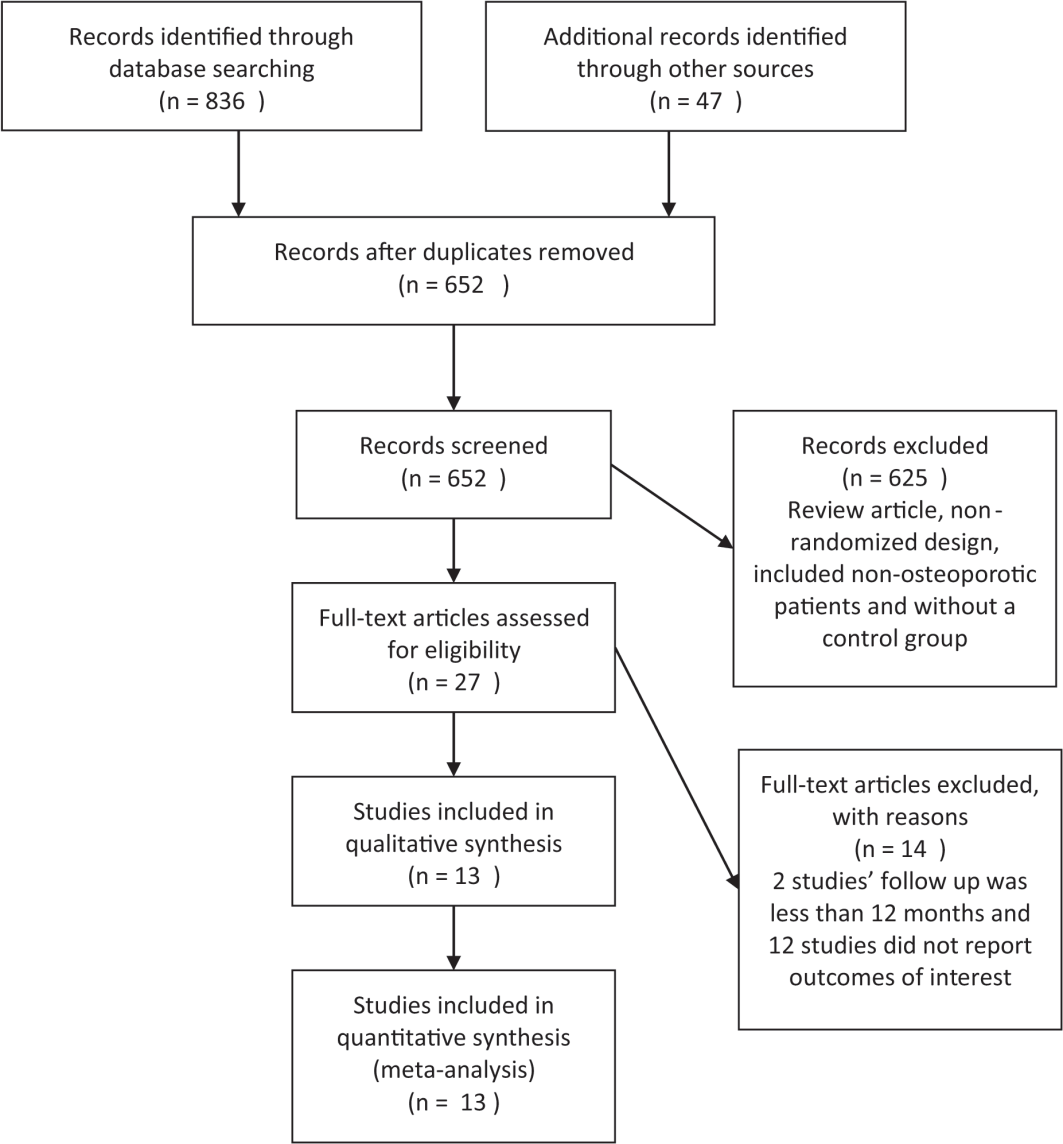
PRISMA flow diagram.

The PRISMA flow diagram of studies is depicted in Fig 1. The last electronic search was performed on May 25^th^, 2014 and identified 836 related references in the primary search and 47 through other sources. After removal of 231 duplicate references, 652 records were screened. Twenty-seven publications were eligible for inclusion criteria, whereas others were not selected for various reasons (e.g., studies without a control group or that included non-osteoporotic patients). A total of 13 studies were included in the qualitative synthesis, and data from these studies were included in the meta-analysis [17–29]. Fourteen studies were excluded: 2 studies due to a follow up time of less than 12 months [30, 31] and 12 studies due to not reporting the outcomes of interest [32–43].

Table 1 provides a summary of the studies included in the review. A total of 3647 participants were included in this meta-analysis. The study sample size ranged from 23 to 1199. These studies were published between 2000 and 2013.

**Table 1:** Study Characteristics.

Trial	Sample size	Comparators	Country	Follow-up	Clinical setting
2000,Orwoll	241	ALE v PLA	Multicenter	24 months	PO
2001,Ringe	134	ALE v ALF	Germany	24 months	PO
2004,Ringe	134	ALE v ALF	Germany	36 months	PO
2009,Boonen	284	RIS v PLA	Multicenter	24 months	PO
2010,Orwoll	135	IBA v PLA	USA	12 months	PO;IO;HO
2010,Orwoll	302	ZOL v ALE	Multicenter	24 months	PO;HO
2012,Boonen	1199	ZOL v PLA	Multicenter	24 months	PO;HO
2010,Ringe	152	STR v ALE	Germany	12 months	PO
2013,Kaufman	261	STR v PLA	Multicenter	24 months	PO
2005,Kaufman	437	TER(20μg) v TER(40μg) v PLA	Multicenter	48 months	IO;HO
2013,Walker	29	RIS v TER(20μg) v Both	USA	18 months	IO
2009,Ringe	316	RIS v PLA	Germany	24 months	PO
2000,Kurland	23	PTH v PLA	USA	18 months	IO

ALE: Alendronate; PLA: Placebo; ALF: Alfacalcidol; RIS: Risedronate; IBA: Ibandronate; ZOL: Zoledronic Acid; STR: Strontium Ranelate; TER: Teriparatide; PTH: Parathyroid Hormone; PO: Primary Osteoporosis; IO: Idiopathic Osteoporosis; HO: Hypogonadal Osteoporosis

### Risk of bias in included studies

**Fig 2:**
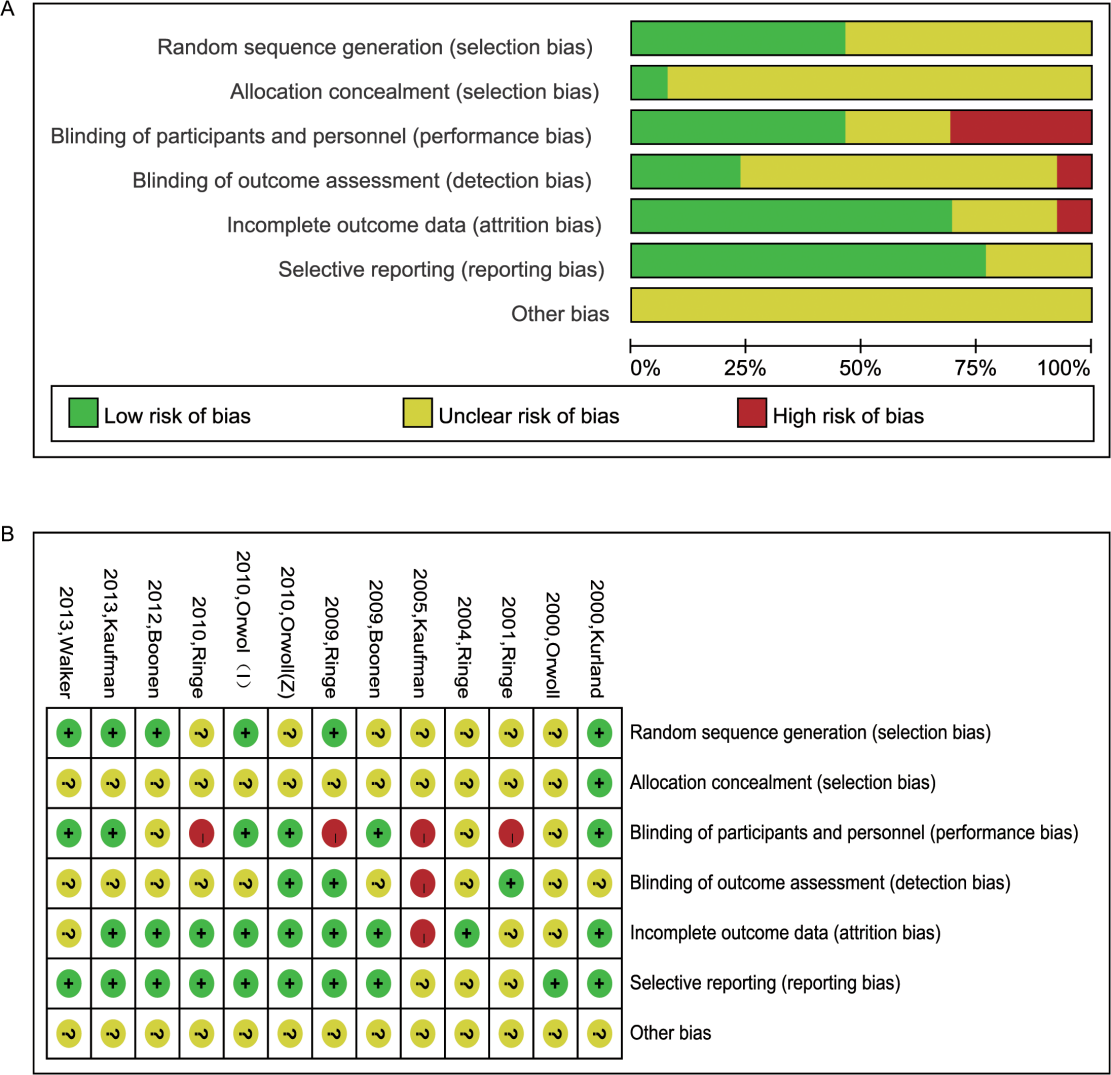
Risk of bias graph and summary. Panel A: Risk of bias graph: review authors’ judgments about each risk of bias item presented as percentages across all included studies. Panel B: Risk of bias summary: review authors’ judgments about each risk of bias item for each included study.

Fig 2 shows the risk of bias in all 13 studies. Six studies described random sequence generation. Only one study described adequate allocation concealment. Seven studies described blinding of participants and personnel. Four studies did not blind to participants and personnel. Three studies described blinding of outcome assessment. One study had a high risk of bias in blinding of outcome assessment. Nine studies had a low risk of incomplete outcome data. One study was considered as a high risk of incomplete outcome data. Ten studies had a low risk of selectively reporting results.

### BMD in LS

**Fig 3:**
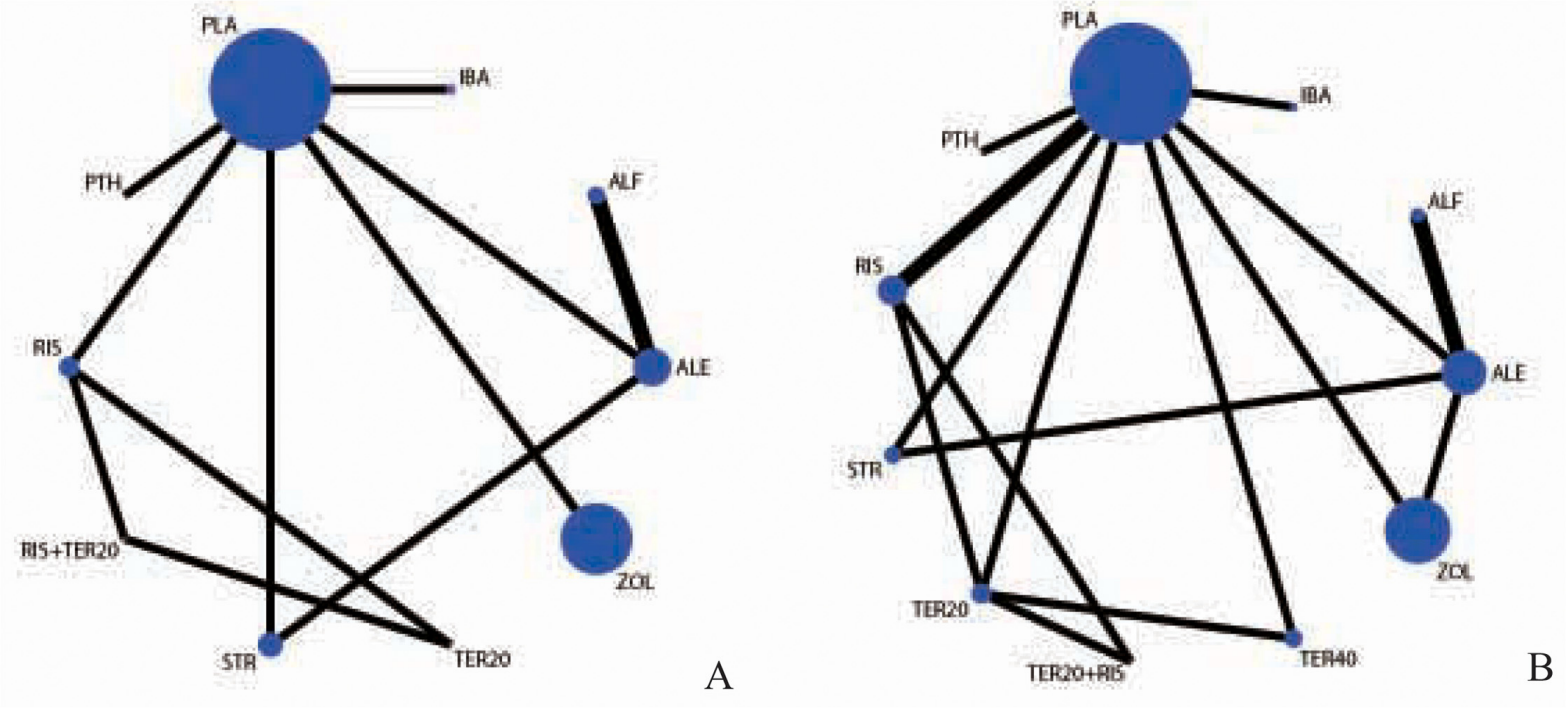
Network of treatment comparisons. The size of the nodes represents the total sample size of treatments. The lines’ thickness corresponds to the number of trials that compare each other. Panel A: Network of treatment comparisons for the BMD in LS. Panel B: Network of treatment comparisons for the fracture rate. ALE: Alendronate; PLA: Placebo; ALF: Alfacalcidol; RIS: Risedronate; IBA: Ibandronate; ZOL: Zoledronate; STR: Strontium Ranelate; TER: Teriparatide; PTH: Parathyroid Hormone.

The network of comparisons on the BMD in LS is shown in Fig 3A. A total of 916 patients were assigned to placebo therapy, 588 to zoledronate therapy, 335 to alendronate therapy, 237 to strontium ranelate therapy, 198 to risedronate therapy, 132 to alfacalcidol therapy, 85 to ibandronate therapy, 13 to parathyroid hormone (1–84) therapy, 10 to teriparatide (20mcg) + risedronate therapy and 9 to teriparatide (20mcg) therapy.

Compared with placebo therapy, zoledronate (SMDs 13.48, 95%CrI 11.88–15.08) yielded the most significant effect on increasing the BMD in LS, followed by alendronate (11.04, 9.68–12.41), teriparatide (20mcg) + risedronate (10.98, 8.55–13.48), risedronate (10.33, 8.68–12.01), teriparatide (20mcg) (9.33, 6.87–11.76), strontium ranelate (8.88, 7.51–10.24), ibandronate (5.49, 3.82–7.16), parathyroid hormone (4.89, 3.12–6.62) and alfacalcidol (3.42, 1.7–5.2). Except for teriparatide (20mcg) + risedronate therapy (2.5, -0.41–5.47), zoledronate therapy was better than other active therapies: 2.44 (0.36–4.55) for alendronate, 10.06 (7.66–12.46) for alfacalcidol, 3.14 (0.83–5.49) for risedronate, 7.98 (5.73–10.28) for ibandronate, 4.6 (2.51–6.72) for strontium ranelate, 4.15 (1.23–7.11) for teriparatide (20mcg) and 8.58 (6.27–10.93) for parathyroid hormone (1–84). Details pertaining to other comparisons are listed in S1 Table. The result of the model test showed that the posterior mean residual deviance (23.73) approximated the data points (21), which confirmed the fitness of the model.

### Fracture rate

The network of comparisons on the fracture rate is shown in Fig 3B. A total of 1142 patients were assigned to placebo therapy, 707 to zoledronate therapy, 506 to alendronate therapy, 353 to risedronate therapy, 196 to strontium ranelate therapy, 132 to alfacalcidol therapy, 101 to teriparatide (20mcg) therapy, 86 to ibandronate therapy, 84 to teriparatide (40mcg) therapy, 10 to parathyroid hormone (1–84) therapy and 10 to teriparatide (20mcg) + risedronate therapy.

The use of placebo therapy resulted in a significantly higher fracture rate in contrast to risedronate (OR 2.51, 95% CrI 1.23–4.24) or zoledronate (2.92, 1.29–5.62) or teriparatide (20mcg) (4.04, 1.36–8.49) or teriparatide (40mcg) (3.5, 1.14–8.34). Alfacalcidol therapy significantly increased the fracture rate compared with risedronate (7.66, 1.74–19.27) or zoledronate (8.41, 2.12–20.03) or strontium ranelate (5.21, 1.32–11.88) or teriparatide (20mcg) (12.12, 2.17–33.84) or teriparatide (40mcg) (10.49, 1.83–30.47). There were no significant differences between other therapies. The details of other comparisons are listed in S2 Table. The result of the model test showed a posterior mean residual deviance (26.23) that approximated the data points (28), which confirmed the fitness of the model.

### Comparisons between traditional pairwise and Bayesian network meta-analyses

**Fig 4:**
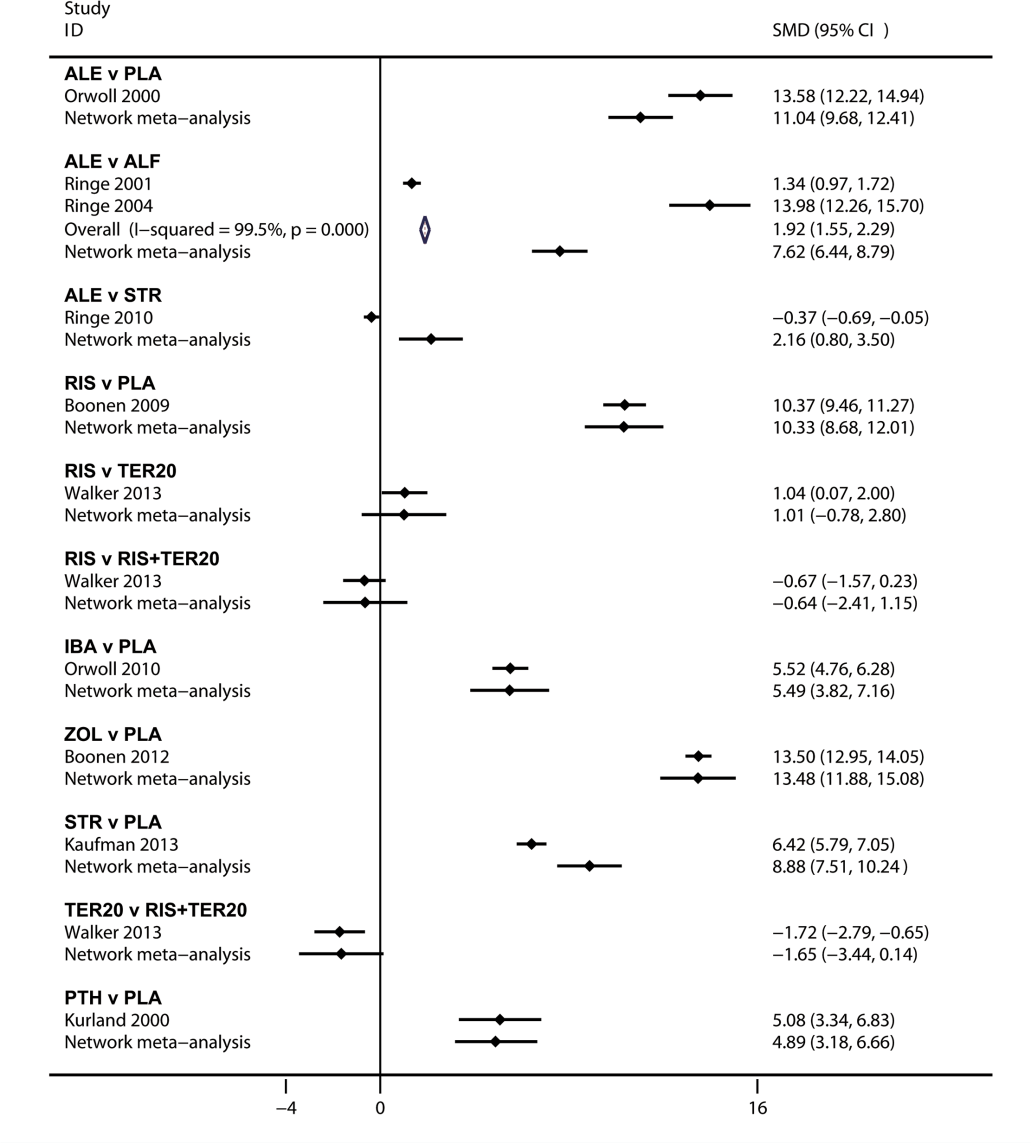
Pooled SMD for the BMD in LS by Bayesian network meta-analysis and traditional meta-analysis. ALE: Alendronate; PLA: Placebo; ALF: Alfacalcidol; RIS: Risedronate; IBA: Ibandronate; ZOL: Zoledronate; STR: Strontium Ranelate; TER: Teriparatide; PTH: Parathyroid Hormone.

The results of the pairwise and Bayesian network meta-analysis are shown in Fig 4 and S1 Fig. The CI from the pairwise meta-analyses and the CrI from the Bayesian network meta-analyses almost overlapped, which indicated that there were no inconsistencies between direct and indirect comparisons.

### Ranking of treatments

**Fig 5:**
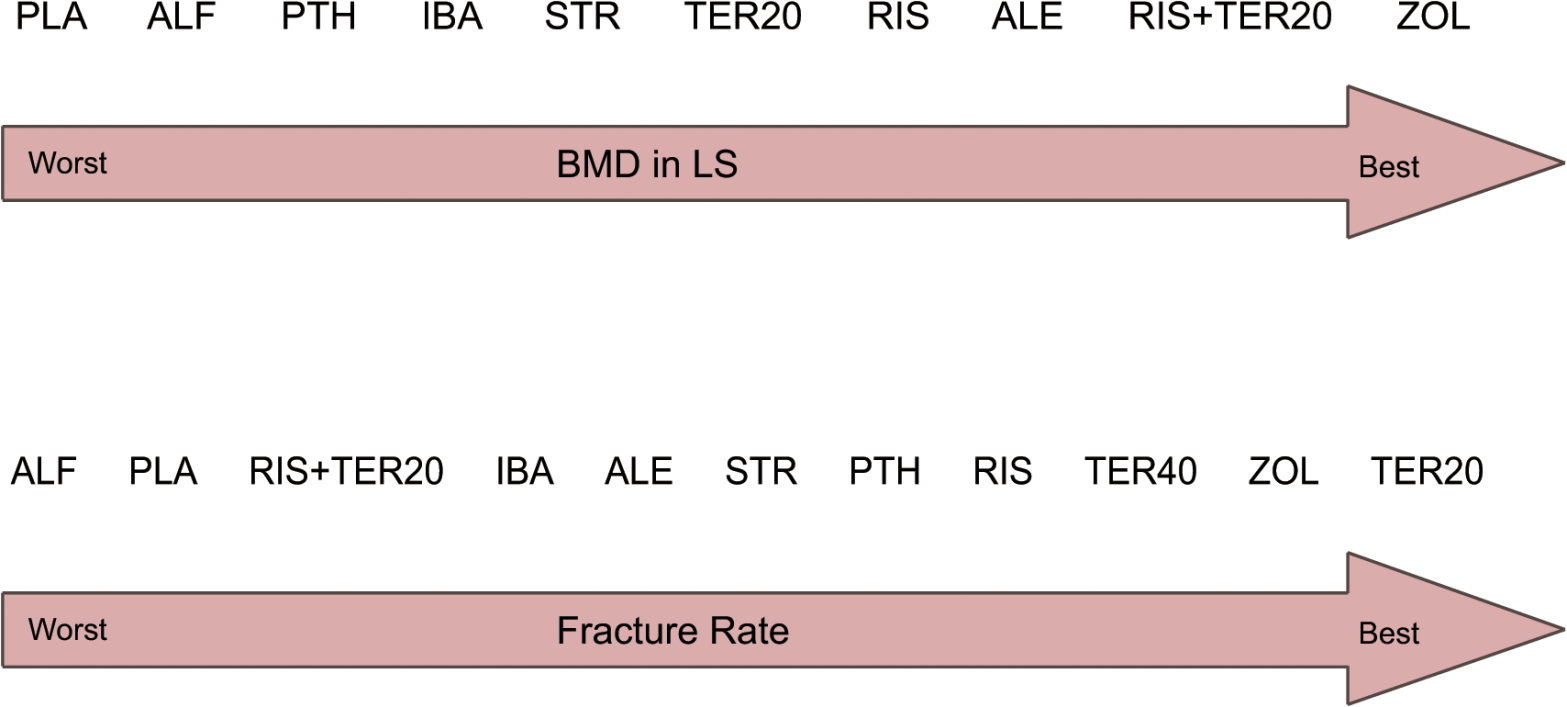
Ranking of treatments in terms of the BMD in LS and fracture rate. ALE: Alendronate; PLA: Placebo; ALF: Alfacalcidol; RIS: Risedronate; IBA: Ibandronate; ZOL: Zoledronate; STR: Strontium Ranelate; TER: Teriparatide; PTH: Parathyroid Hormone.

In Fig 5, we summarized the ranking of eight drugs for eleven treatment strategies in terms of the BMD in LS and fracture rate—with details supplied in S3 Table. For increasing the BMD in LS, zoledronate might be the best therapy and placebo most likely the worst. For decreasing the fracture rate, teriparatide (20mcg) might be the best option, and alfacalcidol ranked the lowest.

### Publication bias and sensitivity analyses

The funnel plots were not performed because the number of included studies in one comparison was less than 10. Overall, the sensitivity analyses (S4 Table and S5 Table) did not change the results.

## Discussion

### Summary of main results

The network meta-analysis provided hierarchies for the BMD in LS and the fracture rate of the different therapies in men with osteoporosis. The meta-analysis indicated that: For increasing the BMD in LS, zoledronate might be the best therapy and placebo might be the worst one. For decreasing the fracture rate, teriparatide (20mcg) might be the best option and alfacalcidol might the worst one.

### Strengths and weaknesses

There are some strengths in this paper: (1) we used a comprehensive search strategy to minimize the possibility of publication bias, (2) we included the result of direct comparisons and indirect comparisons, and (3) we tested the fitness of the model. However, the result of the review should be interpreted under some limitations. First, both the number of the included studies and the sample size were small, which might affect the outcome. For the BMD in LS, zoledronate, ibandronate, teriparatide (20mcg), parathyroid hormone (1–84) and teriparatide (20mcg) + risedronate were analyzed in only one study. Moreover, the sample size for the latter four drugs was less than 100. For the fracture rate, ibandronate, parathyroid hormone (1–84) and teriparatide (20mcg) + risedronate were mentioned in one study and their sample size was less than 100. Therefore, the results presented in this meta-analysis need to be carefully interpreted. Second, some study characteristics such as performance bias and detection bias might be potential interferences for our study. Third, there was substantial heterogeneity due to the inconformity regarding the duration of follow-up. Fourth, most of included studies (61.53%) were placebo-controlled trials that might overestimate the beneficial effect of the active therapies. Fifth, for the studies where the fracture rate was a secondary outcome, the number of measuring time point was insufficient which might underestimate the fracture rate. For example, in one study performed by Ringe et al, the BMD was measured at baseline and at 6, 12, 18, 24 months. The definitive fracture was measured at baseline and at 12, 24 months. Therefore, some fracture events might be missed due to the healing of fracture in 6 months. We should cautiously interpret the results due to the underestimate of the fracture rate to some extent. Sixth, our article used summary data instead of individual patient data, which might lead to the loss of some covariates at the individual patient level. Seventh, due to some hypogonadal men [Two of included studies mentioned the detailed number of the patients and the number of hypogonadal men is small (7.9%, 24/302 in Orwoll 2011; 0.25%, 3/1199 in Boonen 2012)] were including in our article, some potential biases were introduced to our results. The sensitivity analysis by excluding the hypogonadal men could not be performed due to the relevant data could not be extracted. Finally, because four studies had a high risk of bias in blinding of participants and personnel, one study did not blind to outcome assessment and one study had a high risk of bias in incomplete outcome data, the performance, detection and attribution bias might affect the results.

### Agreements and disagreements in the current literature

Prior meta-analyses have mainly focused on postmenopausal osteoporosis or osteoporosis in both males and females; however, less is known about osteoporosis in men. Although previous studies have mixed combinations, the rank methods are relatively rough and their including drugs are not comprehensive [44–46]. Therefore, we performed this network meta-analysis. Only one previous meta-analysis included men with osteoporosis and its results showed that both antiresorptive treatments (alendronate, risedronate, ibandronate, nasal miacalcic and zoledronate) and anabolic treatments (teriparatide) significantly increased spine BMD. For reducing the incidence of fractures, the results are inconclusive [47]. Overall, our results agree with previous research. In addition, our article supports current guidelines to use bisphosphonates (alendronate, risedronate, ibandronate and zoledronate), teriparatide and alfacalcidol in patients with osteoporosis [48, 49] and makes hierarchies of these drugs that previous reviews did not include.

Currently, one RCT for parathyroid hormone has been completed, but data pertaining to this RCT has not been published. Results from this RCT might add new evidence for treating osteoporosis in men [50]. Moreover, data from a completed cohort study with a maximum follow-up duration of 17 years is waiting to be analyzed, and this study investigated the long-term efficacy and tolerability of different treatments in men with osteoporosis (however, no detailed drugs were mentioned) [51].

### Conclusions

This meta-analysis provides evidence that zoledronate might be the best choice to increase the BMD in LS and teriparatide (20mcg) might lead to the lowest fracture rate. Placebo and alfacalcidol might the worst option in increasing the BMD in LS and decreasing the fracture rate, respectively. Higher quality RCTs and direct head to head trials are needed to confirm these results.

## Supporting Information

S1 Fig Pooled odds ratio for fracture rate by Bayesian network meta-analysis and traditional meta-analysis. (EPS)

S1 File A list of full-text excluded articles. (DOC)

S1 PRISMA Checklist PRISMA Checklist for this meta-analysis. (DOC)

S1 Table The BMD in LS for different treatments. (DOC)

S2 Table The fracture rate for different treatments. (DOC)

S3 Table The SUCRA of different therapies in different outcomes. (DOC)

S4 Table Sensitivity analysis: the BMD in LS for different treatments (exclude trials with a high risk of bias). (DOC)

S5 Table Sensitivity analysis: the fracture rate for different treatments (exclude trials with a high risk of bias). (DOC)
