# Supplementary material for: Comparison of Bone Mineral Density in Lumbar Spine and Fracture Rate among Eight Drugs in Treatments of Osteoporosis in Men: A Network Meta-Analysis
Source: PLoS One. 2015 May 26;10(5):e0128032. doi: 10.1371/journal.pone.0128032 (PMC4444106; doi:10.1371/journal.pone.0128032)
Supplement: S1 File — (DOC) [file pone.0128032.s002.doc]

1.Sun Y, Mao M, Sun L, Feng Y, Yang J, et al. (2002) Treatment of osteoporosis in men using dehydroepiandrosterone sulfate. Chin Med J (Engl) 115: 402-404.

**Follow up time was less than 12 months**

2. Hwang JS, Liou MJ, Ho C, Lin JD, Huang YY, et al. (2010) The effects of weekly alendronate therapy in Taiwanese males with osteoporosis. J Bone Miner Metab 28: 328-333.

**Follow up time was less than 12 months**

3. Ringe JD, Dorst A, Kipshoven C, Rovati LC, Setnikar I (1998) Avoidance of vertebral fractures in men with idiopathic osteoporosis by a three year therapy with calcium and low-dose intermittent monofluorophosphate. Osteoporos Int 8: 47-52.

**Not reporting the outcomes of interest**

4. Hamdy RC, Moore SW, Whalen KE, Landy C (1998) Nandrolone decanoate for men with osteoporosis. Am J Ther 5: 89-95.

**Not reporting the outcomes of interest**

5. Ho YV, Frauman AG, Thomson W, Seeman E (2000) Effects of alendronate on bone density in men with primary and secondary osteoporosis. Osteoporos Int 11: 98-101.

**Not reporting the outcomes of interest**

6. Adami S, Prizzi R, Colapietro F (2001) Alendronate for the treatment of osteoporosis in men. Calcif Tissue Int 69: 239-241.

**Not reporting the outcomes of interest**

7. Gillberg P, Mallmin H, Petren-Mallmin M, Ljunghall S, Nilsson AG (2002) Two years of treatment with recombinant human growth hormone increases bone mineral density in men with idiopathic osteoporosis. J Clin Endocrinol Metab 87: 4900-4906.

**Not reporting the outcomes of interest**

8. Finkelstein JS, Hayes A, Hunzelman JL, Wyland JJ, Lee H, et al. (2003) The effects of parathyroid hormone, alendronate, or both in men with osteoporosis. N Engl J Med 349: 1216-1226.

**Not reporting the outcomes of interest**

9. Gonnelli S, Cepollaro C, Montagnani A, Bruni D, Caffarelli C, et al. (2003) Alendronate treatment in men with primary osteoporosis: a three-year longitudinal study. Calcif Tissue Int 73: 133-139.

**Not reporting the outcomes of interest**

10. Orwoll ES, Scheele WH, Paul S, Adami S, Syversen U, et al. (2003) The effect of teriparatide [human parathyroid hormone (1-34)] therapy on bone density in men with osteoporosis. J Bone Miner Res 18: 9-17.

**Not reporting the outcomes of interest**

11. Ringe JD, Faber H, Farahmand P, Dorst A (2006) Efficacy of risedronate in men with primary and secondary osteoporosis: results of a 1-year study. Rheumatol Int 26: 427-431.

**Not reporting the outcomes of interest**

12. Guven Z, Karadag-Saygi E, Unlu-Ozkan F, Akyuz G (2007) The effects of daily alendronate, daily calcitonin and alendronate every other day on bone mineral density in osteoporotic men. Aging Male 10: 197-201.

**Not reporting the outcomes of interest**

13. Majima T, Shimatsu A, Komatsu Y, Satoh N, Fukao A, et al. (2008) Efficacy of risedronate in Japanese male patients with primary osteoporosis. Intern Med 47: 717-723.

**Not reporting the outcomes of interest**

14. Boonen S, Lorenc RS, Wenderoth D, Stoner KJ, Eusebio R, et al. (2012) Evidence for safety and efficacy of risedronate in men with osteoporosis over 4 years of treatment: Results from the 2-year, open-label, extension study of a 2-year, randomized, double-blind, placebo-controlled study. Bone 51: 383-388.

**Not reporting the outcomes of interest**
