# Supplementary material for: Comparison of Bone Mineral Density in Lumbar Spine and Fracture Rate among Eight Drugs in Treatments of Osteoporosis in Men: A Network Meta-Analysis
Source: PLoS One. 2015 May 26;10(5):e0128032. doi: 10.1371/journal.pone.0128032 (PMC4444106; doi:10.1371/journal.pone.0128032)
Supplement: S1 Table — (DOC) [file pone.0128032.s004.doc]

| ALE | **11.04**  **[9.679,12.41]** | **7.621**  **[6.437,8.794]** | 0.7051  [-1.458,2.92] | **5.545**  **[3.411,7.681]** | **-2.439**  **[-4.553,-0.3636]** | **2.157**  **[0.8002,3.5]** | 1.711  [-1.141,4.56] | 0.06126  [-2.805,2.878] | **6.144**  **[3.942,8.321]** |
| --- | --- | --- | --- | --- | --- | --- | --- | --- | --- |
| **13.576**  **[12.216,14.936]** | PLA | **-3.417**  **[-5.198,-1.663]** | **-10.33**  **[-12.01,-8.678]** | **-5.494**  **[-7.155,-3.815]** | **-13.48**  **[-15.08,-11.88]** | **-8.881**  **[-10.24,-7.511]** | **-9.328**  **[-11.76,-6.871]** | **-10.98**  **[-13.48,-8.55]** | **-4.894**  **[-6.622,-3.175]** |
| 7.633  [-4.75,20.015] | / | ALF | **-6.916**  **[-9.39,-4.395]** | -2.077  [-4.529,0.3602] | **-10.06**  **[-12.46,-7.658]** | **-5.464**  **[-7.265,-3.712]** | **-5.911**  **[-9.031,-2.846]** | **-7.56**  **[-10.67,-4.461]** | -1.477  [-3.962,1.019] |
| / | **-10.369**  **[-11.275,-9.463]** | / | RIS | **4.84**  **[2.486,7.195]** | **-3.144**  **[-5.488,-0.8289]** | 1.452  [-0.7059,3.609] | 1.006  [-0.7799,2.795] | -0.6438  [-2.409,1.145] | **5.439**  **[3.048,7.853]** |
| / | **-5.524**  **[-6.284,-4.764]** | / | / | IBA | **-7.984**  **[-10.28,-5.731]** | **-3.387**  **[-5.534,-1.236]** | **-3.834**  **[-6.799,-0.9004]** | **-5.484**  **[-8.449,-2.54]** | 0.5993  [-1.813,3.02] |
| / | **-13.501**  **[-14.054,-12.949]** | / | / | / | ZOL | **4.597**  **[2.505,6.719]** | **4.15**  **[1.231,7.113]** | 2.5  [-0.4085,5.474] | **8.583**  **[6.269,10.93]** |
| **-0.366**  **[-0.687,-0.045]** | **-6.417**  **[-7.048,-5.785]** | / | / | / | / | STR | -0.4468  [-3.312,2.358] | -2.096  [-4.972,0.7524] | **3.987**  **[1.8, 6.196]** |
| / | / | / | **1.036**  **[0.070, 2.001]** | / | / | / | TER20 | -1.649  [-3.44,0.1395] | **4.433**  **[1.412,7.488]** |
| / | / | / | -0.668  [-1.572,0.235] | / | / | / | **-1.721**  **[-2.791,-0.651]** | RIS+TER20 | **6.083**  **[3.147,9.121]** |
| / | **-5.084**  **[-6.829,-3.339]** | / | / | / | / | / | / | / | PTH |

S1 Table. The BMD in LS for different treatments.

For the BMD in LS, standard mean differences (SMDs) lower than 0 favored the column-defining treatment. Direct comparisons were shown in the bottom left. Indirect comparisons were shown in the upper right. The number which was painted by a style of overstriking indicated there was a significant difference between two treatments. ALE: Alendronate; PLA: Placebo; ALF: Alfacalcidol; RIS: Risedronate; IBA: Ibandronate; ZOL: Zoledronate; STR: Strontium Ranelate; TER20: Teriparatide 20mg; RIS+TER20: Risedronate + Teriparatide 20mg; PTH: Parathyroid Hormone.
