# Supplementary material for: Comparison of Bone Mineral Density in Lumbar Spine and Fracture Rate among Eight Drugs in Treatments of Osteoporosis in Men: A Network Meta-Analysis
Source: PLoS One. 2015 May 26;10(5):e0128032. doi: 10.1371/journal.pone.0128032 (PMC4444106; doi:10.1371/journal.pone.0128032)
Supplement: S2 Table — (DOC) [file pone.0128032.s005.doc]

S2 Table. The fracture rate for different treatments.

| ALE | 0.9406  [0.4411, 1.751] | **0.3794**  **[0.1681, 0.7194]** | 15.09  [0.08454, 31.38] | 1.82  [0.04781, 7.126] | 2.363  [0.7796, 5.016] | 1.411  [0.09796, 4.464] | 2.606  [0.9945, 5.332] | 1.614  [0.5954, 3.264] | 3.759  [0.9553, 9.378] | 3.309  [0.784, 8.87] |
| --- | --- | --- | --- | --- | --- | --- | --- | --- | --- | --- |
| 0.940  [0.416，2.124] | PLA | 0.4895  [0.1435, 1.091] | 19.52  [0.1127, 32.52] | 1.937  [0.06517, 7.626] | **2.508**  **[1.232, 4.238]** | 1.489  [0.1365, 4.216] | **2.923**  **[1.292, 5.619]** | 1.913  [0.6634, 4.202] | **4.04**  **[1.355, 8.493]** | **3.495**  **[1.138, 8.335]** |
| **0.358**  **[0.173，0.740]** | / | ALF | 38.02  [0.2504, 88.15] | 5.722  [0.1299, 22.0] | **7.658**  **[1.741, 19.27]** | 4.613  [0.2345, 15.65] | **8.407**  **[2.12, 20.03]** | **5.21**  **[1.32, 11.88]** | **12.12**  **[2.167, 33.84]** | **10.49**  **[1.831, 30.47]** |
| / | 1.125  [0.089，14.202] | / | PTH | 4.825  [0.009709, 19.19] | 14.02  [0.06335, 22.62] | 5.804  [0.0161, 12.87] | 14.45  [0.07234, 30.19] | 9.391  [0.04402, 14.66] | 18.65  [0.0961, 34.02] | 12.9  [0.0842, 31.91] |
| / | / | / | / | RIS+TER20 | 9.079  [0.2975, 33.17] | 5.926  [0.04738, 25.84] | 11.87  [0.3019, 44.15] | 8.13  [0.1765, 28.01] | 14.7  [0.4228, 57.61] | 13.57  [0.3485, 53.13] |
| / | **2.312**  **[1.108，4.824]** | / | / | 2.250  [0.170，29.767] | RIS | 0.696  [0.05165, 2.032] | 1.362  [0.4324, 2.966] | 0.8878  [0.2334, 2.043] | 1.849  [0.4785, 4.3] | 1.616  [0.415, 4.3] |
| / | 0.911  [0.161，5.170] | / | / | / | / | IBA | 7.141  [0.5584, 23.52] | 4.802  [0.2956, 15.48] | 8.867  [0.6458, 30.36] | 8.819  [0.5208, 31.53] |
| 1.585  [0.438，5.732] | **3.100**  **[1.449，6.630]** | / | / | / | / | / | ZOL | 0.7814  [0.2072, 1.826] | 1.709  [0.3949, 4.181] | 1.483  [0.3402, 4.004] |
| 1.494  [0.537，4.155] | 1.368  [0.416，4.497] | / | / | / | / | / | / | STR | 2.841  [0.6225, 7.685] | 2.499  [0.4295, 6.904] |
| / | **3.242**  **[1.234，8.517]** | / | / | 5.588  [0.234，133.607] | 3.000  [0.108，83.359] | / | / | / | TER20 | 1.086  [0.2905, 2.727] |
| / | **2.940**  **[1.117，7.743]** | / | / | / | / | / | / | / | 0.907  [0.281，2.929] | TER40 |

For the fracture rate, odds ratios (ORs) higher than 1 favored the column-defining treatment. Direct comparisons were shown in the bottom left. Indirect comparisons were shown in the upper right. The number which was painted by a style of overstriking indicated there was a significant difference between two treatments. ALE: Alendronate; PLA: Placebo; ALF: Alfacalcidol; RIS: Risedronate; IBA: Ibandronate; ZOL: Zoledronate; STR: Strontium Ranelate; TER20: Teriparatide 20mg; TER40: Teriparatide 40mg; RIS+TER20: Risedronate + Teriparatide 20mg; PTH: Parathyroid Hormone.
