# Supplementary material for: Comparison of Bone Mineral Density in Lumbar Spine and Fracture Rate among Eight Drugs in Treatments of Osteoporosis in Men: A Network Meta-Analysis
Source: PLoS One. 2015 May 26;10(5):e0128032. doi: 10.1371/journal.pone.0128032 (PMC4444106; doi:10.1371/journal.pone.0128032)
Supplement: S3 Table — (DOC) [file pone.0128032.s006.doc]

S3 Table. The SUCRA of different therapies in different outcomes.

|  | ALE | PLA | ALF | PTH | RIS+TER20 | RIS | IBA | ZOL | STR | TER20 | TER40 |
| --- | --- | --- | --- | --- | --- | --- | --- | --- | --- | --- | --- |
| BMD in LS | 0.2142 | 0.9999 | 0.8619 | 0.757 | 0.2097 | 0.3023 | 0.711 | 0.01468 | 0.4839 | 0.4453 | NA |
| FRA | 0.3774 | 0.2973 | 0.08691 | 0.5851 | 0.3071 | 0.6771 | 0.3677 | 0.7309 | 0.5349 | 0.8153 | 0.7203 |

For the BMD (bone mineral density) in LS (lumbar spine), the smallest value meant ranking the first; for the FRA (fracture rate), the largest value meant ranking the first.

ALE: Alendronate; PLA: Placebo; ALF: Alfacalcidol; RIS: Risedronate; IBA: Ibandronate; ZOL: Zoledronate; STR: Strontium Ranelate; TER20: Teriparatide 20mg; TER40: Teriparatide 40mg; RIS+TER20: Risedronate + Teriparatide 20mg; PTH: Parathyroid Hormone.
