# Supplementary material for: Comparison of Bone Mineral Density in Lumbar Spine and Fracture Rate among Eight Drugs in Treatments of Osteoporosis in Men: A Network Meta-Analysis
Source: PLoS One. 2015 May 26;10(5):e0128032. doi: 10.1371/journal.pone.0128032 (PMC4444106; doi:10.1371/journal.pone.0128032)
Supplement: S4 Table — (DOC) [file pone.0128032.s007.doc]

S4 Table. Sensitivity analysis: the BMD in LS for different treatments (exclude trials with a high risk of bias).

| ALE | **13.51**  **[12.57,14.42]** | **13.9**  **[12.95,14.85]** | **3.163**  **[1.829,4.485]** | **8.01**  **[6.681,9.366]** | 0.027  [-1.282,1.307] | **7.124**  **[5.814,8.462]** | **4.131**  **[2.457,5.889]** | **2.483**  **[0.8139,4.219]** | **8.591**  **[7.177,9.971]** |
| --- | --- | --- | --- | --- | --- | --- | --- | --- | --- |
| **/** | PLA | 0.3903  [-0.913,1.749] | **-10.35**  **[-11.29,-9.4]** | **-5.502**  **[-6.44,-4.54]** | **-13.49**  **[-14.39,-12.57]** | **-6.388**  **[-7.318,-5.442]** | **-9.381**  **[-10.83,-7.85]** | **-11.03**  **[-12.5,-9.52]** | **-4.92**  **[-5.98,-3.84]** |
| / | / | ALF | **-10.74**  **[-12.37,-9.14]** | **-5.89**  **[-7.56,-4.2]** | **-13.88**  **[-15.51,-12.24]** | **-6.78**  **[-8.4,-5.1]** | **-9.77**  **[-11.73,-7.8]** | **-11.42**  **[-13.34,-9.42]** | **-5.31**  **[-7,1.-3.61]** |
| / | **/** | / | RIS | **4.846**  **[3.49,6.2]** | **-3.137**  **[-4.44,-1.84]** | **3.961**  **[2.63,5.35]** | 0.9674  [-0.15,2.15] | -0.6805  [-1.82,0.45] | **5.427**  **[4,6.85]** |
| / | **/** | / | / | IBA | **-7.983**  **[-9.3,-6.67]** | -0.8856  [-2.215,0.45] | **-3.879**  **[-5.59,-2.04]** | **-5.527**  **[-7.23,-3.74]** | 0.5807  [-0.86,1.99] |
| / | **/** | / | / | / | ZOL | **7.098**  **[5.81,7.39]** | **4.104**  **[2.39,5.898]** | **2.456**  **[0.76,4.2]** | **8.564**  **[7.15,9.95]** |
| **/** | **/** | / | / | / | / | STR | **-2.993**  **[-4.725,-1.16]** | **-4.64**  **[-6.43,-2.88]** | **1.466**  **[0.04, 2.85]** |
| / | / | / | **/** | / | / | / | TER20 | **-1.648**  **[-2.79,-0.52]** | **4.46**  **[2.57,6.24]** |
| / | / | / | / | / | / | / | **/** | RIS+TER20 | **6.108**  **[4.25,7.94]** |
| / | **/** | / | / | / | / | / | / | / | PTH |

For the BMD in LS, standard mean differences (SMDs) lower than 0 favored the column-defining treatment. Indirect comparisons were shown in the upper right. The number which was painted by a style of overstriking indicated there was a significant difference between two treatments. ALE: Alendronate; PLA: Placebo; ALF: Alfacalcidol; RIS: Risedronate; IBA: Ibandronate; ZOL: Zoledronate; STR: Strontium Ranelate; TER20: Teriparatide 20mg; RIS+TER20: Risedronate + Teriparatide 20mg; PTH: Parathyroid Hormone.
