# Supplementary material for: Comparison of Bone Mineral Density in Lumbar Spine and Fracture Rate among Eight Drugs in Treatments of Osteoporosis in Men: A Network Meta-Analysis
Source: PLoS One. 2015 May 26;10(5):e0128032. doi: 10.1371/journal.pone.0128032 (PMC4444106; doi:10.1371/journal.pone.0128032)
Supplement: S5 Table — (DOC) [file pone.0128032.s008.doc]

S5 Table. Sensitivity analysis: the fracture rate for different treatments (exclude trials with a high risk of bias).

| ALE | 0.9649  [0.3123, 2.055] | 0.4367  [0.1082, 1.025] | 8.068  [0.1143, 31.42] | 1.448  [0.028, 5.34] | 1.844  [0.2428, 5.564] | 1.406  [0.088, 4.62] | 2.787  [0.7861, 5.424] | 1.815  [0.2259, 3.264] | 4.034  [0.6613, 11.74] | 3.879  [0.5764, 11.22] |
| --- | --- | --- | --- | --- | --- | --- | --- | --- | --- | --- |
| / | PLA | 0.6419  [0.099, 1.815] | 8.881  [0.1783, 35.33] | 1.349  [0.0397, 4.953] | 1.855  [0.4126, 4.708] | 1.453  [0.1363, 4.359] | **3.228**  **[1.124, 6.627]** | 1.933  [0.3556, 5.264] | **4.146**  **[1.159, 10.27]** | **3.963**  **[1.138, 8.335]** |
| **/** | / | ALF | 31.29  [0.2527, 118.4] | 6.144  [0.0645, 19.85] | 7.024  [0.509, 24.85] | 5.405  [0.2345, 15.65] | **10.85**  **[1.428, 30.72]** | 7.512  [0.468, 22.79] | **16.11**  **[1.28, 50.21]** | **15.05**  **[1.14, 50.55]** |
| / | / | / | PTH | 2.532  [0.0046, 8.628] | 2.481  [0.029, 10.16] | 2.031  [0.014, 7.74] | 4.523  [0.069, 17.78] | 2.782  [0.029, 10.17] | 6.102  [0.083, 21.96] | 6.145  [0.07, 24.28] |
| / | / | / | / | RIS+TER20 | 10.23  [0.2857, 30.49] | 13.06  [0.09, 34.66] | 26.64  [0.48, 84.83] | 19.53  [0.1767, 62.03] | 26.65  [0.5775, 88.64] | 31.36  [0.4923, 97.57] |
| / | **/** | / | / | / | RIS | 1.411  [0.072, 4.58] | 3.026  [0.4307, 8.659] | 1.872  [0.1561, 6.192] | 3.601  [0.491, 10.65] | 3.53  [0.44, 11.27] |
| / | / | / | / | / | / | IBA | 7.196  [0.508, 23.3] | 4.508  [0.208, 13.5] | 10.01  [0.505, 33.37] | 9.745  [0.4707, 34.37] |
| / | **/** | / | / | / | / | / | ZOL | 0.8172  [0.097, 2.386] | 1.785  [0.3077, 5.133] | 1.72  [0.2563, 5.147] |
| / | / | / | / | / | / | / | / | STR | 4.505  [0.4259, 13.94] | 4.331  [0.3707, 13.62] |
| / | **/** | / | / | / | / | / | / | / | TER20 | 1.282  [0.255, 3.331] |
| / | **/** | / | / | / | / | / | / | / | / | TER40 |

For the fracture rate, odds ratios (ORs) higher than 1 favored the column-defining treatment. Indirect comparsions were shown in the upper right. The number which was painted by a style of overstriking indicated there was a significant difference between two treatments. ALE: Alendronate; PLA: Placebo; ALF: Alfacalcidol; RIS: Risedronate; IBA: Ibandronate; ZOL: Zoledronate; STR: Strontium Ranelate; TER20: Teriparatide 20mg; TER40: Teriparatide 40mg; RIS+TER20: Risedronate + Teriparatide 20mg; PTH: Parathyroid Hormone.
